# Does depression moderate the relationship between pain and suicidality in adolescence? A moderated network analysis

**DOI:** 10.1016/j.jad.2021.05.100

**Published:** 2021-09-01

**Authors:** Verena Hinze, Tamsin Ford, Catherine Crane, Jonas M.B. Haslbeck, Keith Hawton, The MYRIAD Team, Bergljot Gjelsvik

**Affiliations:** a Oxford Mindfulness Centre, Department of Psychiatry, University of Oxford, Oxford, UK; b Department of Psychiatry, University of Cambridge, Cambridge, UK; c Psychological Methods Group, University of Amsterdam, the Netherlands; d Centre for Suicide Research, Department of Psychiatry, University of Oxford, Oxford, UK; e Oxford Health NHS Foundation Trust, Warneford Hospital, Oxford, UK; f Department of Psychology, University of Oslo, Oslo, Norway

**Keywords:** Adolescence, Depression, Network analysis, Pain, Self-harm, Suicidality

## Abstract

**Background:**

Whilst growing research suggests that pain is associated with suicidality in adolescence, it remains unclear whether this relationship is moderated by co-morbid depressive symptoms. The present study aimed to investigate whether the pain-suicidality association is moderated by depressive symptoms.

**Methods:**

We performed secondary analyses on cross-sectional, pre-intervention data from the ‘My Resilience in Adolescence’ [MYRIAD] trial (ISRCTN ref: 86619085; *N*=8072, 11-15 years). Using odds ratio tests and (moderated) network analyses, we investigated the relationship between pain and suicidality, after controlling for depression, anxiety, inhibitory control deficits and peer problems. We investigated whether depression moderates this relationship and explored gender differences.

**Results:**

Overall, 20% of adolescents reported suicidality and 22% reported pain, whilst nine percent of adolescents reported both. The experience of pain was associated with a four-fold increased risk of suicidality and vice versa (*OR*=4.00, 95%-CI=[3.54;4.51]), with no gender differences. This cross-sectional association remained significant after accounting for depression, anxiety, inhibitory control deficits and peer problems (*aOR*=1.39). Depression did not moderate the pain-suicidality association.

**Limitations:**

The item-based, cross-sectional assessment of pain and suicidality precludes any conclusions about the direction of the effects and which aspects of suicidality and pain may drive this association.

**Conclusions:**

Our findings underscore the need to consider pain as an independent risk correlate of suicidality in adolescents. Longitudinal research should examine how this relationship develops during adolescence. Clinically, our findings emphasise the need to assess and address suicidality in adolescents with pain, even in the absence of depressive symptoms.

## Highlights

• One-in-five adolescents reported suicidality or pain.
• Pain was associated with an increased risk of suicidality and vice versa (OR=4.00).
• Network analysis supported the pain-suicidality association (aOR=1.39).
• This cross-sectional association was not moderated by depression.
• Pain should be considered as a key risk correlate of suicidality in adolescents.

## 1 Introduction

Thoughts about suicide and self-harm are a serious public health concern in adolescents (Bridge et al., 2006; World Health Organisation; 2019). About 30 percent of adolescents in the general population report these thoughts, and of those about a third will ultimately enact these thoughts (‘*self-harm*’, defined as non-fatal intentional self-poisoning or self-injury irrespective of suicidal intent or other motives; Evans et al., 2005; Hawton et al., 2003). This definition is used in preference to the categorical separation into non-suicidal self-injury and attempted suicide, as suicidal intent is widely viewed as a dimensional phenomenon (Hawton et al., 2012; Kapur et al., 2013). Self-harm tends to reoccur and is the most important risk factor for future suicide during adolescence (Gillies et al., 2018; Hawton et al., 2020). We apply the term ‘suicidality’ to refer to the broad spectrum of suicidal distress, ranging from thoughts about suicide and self-harm to the enactment of these thoughts (Bridge et al., 2006; O'Connor and Kirtley, 2018). Enhanced knowledge about correlates of suicidality in adolescence is crucial to improve targeted care.

An increasing body of research suggests that physical pain (‘*pain*’) is an important correlate of suicidality in adolescents (Dean-Boucher et al., 2020; Hinze et al., 2019). Pain is highly prevalent in young people (11-38%), especially headaches and abdominal pain (King et al., 2011). These prevalence rates tend to particularly increase during early adolescence, with epidemiological research showing that up to 44 percent of adolescents in schools across 42 countries reported weekly pain over the last six months (Gobina et al., 2019; Martin et al., 2007). If poorly managed, the experience of pain may persist beyond the years of adolescence and significantly impact the young person's life (Brattberg, 2004; Eccleston et al., 2020; Lewandowski Holley et al., 2017), as shown in the Global Burden of Disease Study where headache disorder was highlighted as the second major cause of disability in adolescents worldwide (GBD 2019 Diseases and Injuries Collaborators, 2020). As pain is officially defined as a sensory and emotional experience (Raja et al., 2020), pain may interact with mental health problems, including depression and at its worst suicidality, leading to comorbidities and possibly mutual maintenance relationships between pain and mental health (Soltani et al., 2019). Indeed, similarly to the developmental trajectories of pain, prevalence rates of suicidality tend to increase substantially from the age of 12 years onwards (Nock et al., 2013). Girls are at greater risk of both pain in various locations and self-harm, whilst boys are at an increased risk of completed suicide (Hawton et al., 2012; King et al., 2011).

Other known correlates of suicidality in adolescence involve (i) socio-demographic and educational factors, (ii) stressful life events (e.g., peer problems), and (iii) mental-health factors, especially depressive symptoms and cognitive processes (e.g., problem-solving and impulsivity; Hawton et al., 2012), which largely rely on an individual's inhibitory control – the ability to regulate one's thoughts, attention, behaviours and emotions (Diamond, 2013).

**Fig. 1:**
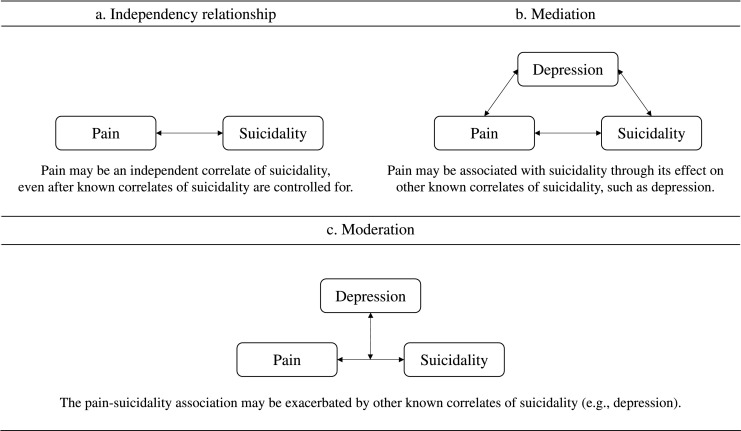
Representation of the potential pain-suicidality association in adolescence.

Currently, it is unknown how correlates of suicidality contribute to the observed relationship between pain and suicidality in adolescence (Fig. 1). Prior research suggests that pain may be an independent correlate of suicidality, after depression and other correlates of suicidality are controlled for (Hinze et al., 2021; Van Tilburg et al., 2011). Alternatively, pain may be associated with suicidality through its effect on depression and other correlates of suicidality (‘mediation’; Soltani et al., 2019). Finally, other correlates (e.g., depression) may exacerbate the effects of pain on suicidality (‘moderation’; Hinze et al., 2019).

To gain knowledge about these complex relationships, we employed statistical network models, which allow us to establish the role of other correlates, identify possible moderation effects and obtain an indication of potential mediation effects in the cross-sectional relationship between pain and suicidality in adolescents aged 11 to 15 years (Haslbeck and Waldorp, 2020). We tested two key hypotheses and explored gender differences:

First, that pain would be associated with suicidality, and that this cross-sectional association would remain significant after accounting for depressive symptoms, anxiety symptoms, peer problems and inhibitory control deficits.

Second, that the pain-suicidality association would be moderated by depression, after accounting for key correlates of suicidality (i.e., anxiety, peer problems and inhibitory control deficits), with a stronger pain-suicidality association in adolescents who reported depressive symptoms.

## 2 Methods

### 2.1 Study design and participants

We performed secondary data analyses, using cross-sectional, pre-intervention data (i.e., following randomisation and prior to the intervention) from the ‘My Resilience in Adolescence’ [MYRIAD] trial (Kuyken et al., 2017). This cluster-randomised controlled trial aims to evaluate the effectiveness of a school-based mindfulness training to improve the mental health and well-being of young people across 85 schools in the United Kingdom (ISRCTN ref: 86619085; Kuyken et al., 2017). Pre-intervention data were collected from two cohorts comprising 84 schools in the academic years 2017/2018 (Cohort 1: *n*=923; 11.4%) and 2018/2019 (Cohort 2: *n*=7149; 88.6%). All pupils were eligible for study participation except if they could not provide informed assent and were unable to understand English. In exceptional circumstances, pupils were excluded, based on school judgement of their inability to participate. Recruitment strategies and additional trial information has been described in Supplement 1. The MYRIAD trial has been granted ethical approval by the University of Oxford Medical Sciences Division Ethics Committee (R45358). Apart from a data access permission, no further approvals were required for the present study. Informed consent was obtained from schools, parents (via opt-out) and pupils prior to participation in this study.

### 2.2 Measures

#### 2.2.1 Sociodemographic data

Participants were asked to report their age, gender and their ethnicity.

#### 2.2.2 Suicidality

We used three standardised questions to assess suicidality via self-report: a) “Have you thought that life was not worth living, or that you would be better off dead?”, b) “Have you thought seriously about trying to harm yourself in some way (for example by cutting yourself or taking an overdose of pills or other medication)?” and c) “Have you actually, deliberately harmed yourself in some way (for example by cutting yourself or taking an overdose of pills or other medication)?” (Kuyken et al., 2017). These questions referred to the past 12 months, with the response options: ‘Yes’, ‘No’ and ‘Prefer not to say’. Given the low prevalence rates of self-harm in our study, we collapsed these items into a single, binary ‘suicidality’ variable. This was coded as present, if at least one item was answered with ‘Yes’, and absent for both other responses.

#### 2.2.3 Pain

The following item of the Strengths and Difficulties Questionnaire ([SDQ]; Goodman, 1997) was used to assess the presence or absence of pain in the past six months: “*I get a lot of headaches, stomach-aches or sickness*”. Pain was coded as absent, if participants selected ‘Not true’, whilst ‘Somewhat True’ and ‘Certainly True’ were coded as pain being present.

The presence of pain on the assessment day was established with the Child Health Utility 9D ([CHU-9D]; Stevens, 2011), including the item: “*Are you in pain today?*” scored from 0 (“I don't have any pain today”) to 4 (“I have a lot of pain today”). Pain was coded as absent if the participant selected ‘I don't have any pain today’ and as present for all other response options. The validity of the overall scale and the specific pain item has been established in adolescents in the general population and clinical settings (Chen et al., 2015; Furber and Segal, 2015; Stevens and Ratcliffe, 2012).

Despite the different reference points (SDQ: 6 months vs. CHU-9D: today), both items were moderately correlated, with a correlation in the expected direction (Spearman's rho=.29, Bootstrapped 95%-CI=[0.27-0.31]). Consistent with previous research, these findings suggest that both items measure the same construct ‘pain’ (i.e., convergent validity; Furber and Segal, 2015). For the present analyses, we created a combined ‘*pain*’ variable. This was coded as present, if adolescents reported both pain in the past six months (SDQ: pain present) and pain on the assessment day (CHU-9D: pain present). If participants reported pain for only one measure (SDQ *or* CHU-9D), or no pain at all, we coded pain as absent. We used this approach to identify adolescents for whom pain may be more pronounced and potentially persistent or recurrent, in contrast to normative pains.

#### 2.2.4 Depression

The Center for Epidemiologic Studies Depression Scale ([CES-D]; Radloff, 1977, 1991) is a well-established valid and reliable screening instrument for self-reported depressive symptoms in epidemiological research with adolescents (Dierker et al., 2001; Garrison et al., 1991; Radloff, 1991; Roberts et al., 1990). Participants were asked to score the 20 symptom items on a four-point scale, ranging from 0 (“Rarely or none of the time (less than 1 day)”) to 3 (“Most or all of the time (5-7 days”)), with a higher score reflecting greater symptom severity. Consistent with the official scoring guidelines (Radloff, 1991), we treated ‘depression’ as an ordinal variable, consisting of the following three categories: normal (score: 0-15), at risk (score: 16-27) and caseness (score: 28-60; Radloff, 1991).

#### 2.2.5 Additional correlates of suicidality

Other measured correlates of suicidality included anxiety (Revised Children's Anxiety and Depression Scale; Chorpita et al., 2000), peer problems and inhibitory control deficits (both measured with the SDQ; Goodman, 1997). We have selected these covariates given their strong empirical support in the context of suicidality and pain (see Hawton et al., 2012; McKillop and Banez, 2016). All correlates were treated as ordinal, categorical variables, with three (anxiety) and four levels (peer problems and inhibitory control deficits). We did not control for further demographic factors as most young people were of similar age (93% aged 12 or 13 years), and as gender differences were explored more thoroughly by comparing the separate networks of boys and girls. Noteworthy, additional exploratory analyses, controlling for age, gender and ethnicity in the network models, provided similar estimates. Further information on the measurements is provided in Supplement 2.

### 2.3 Statistical analyses

Statically analyses were performed in R, version 3.6.2 (R Core Team, 2019) with *p*<0.05 reflecting statistical significance.

#### 2.3.1 Data exploration

We explored sample characteristics using descriptive statistics (R-packages: *psych* version 2.0.12; Revelle, 2020; and *Hmisc* version 4.2-2; Harrell and Dupont, 2020). Gender differences were explored using the Pearson's Chi-squared test for equal proportions (binary data) and the two-sample Wilcoxon test (ordinal data; R-package *stats*, version 3.6.2; R Core Team, 2019). We investigated the pain-suicidality association using graphical visualisations (R-package *ggplot2* version 3.3.3; Wickham et al., 2020) and odds ratio tests (R-package *questionr* version 0.7.4; Barnier et al., 2020).

#### 2.3.2 Rationale for using network analyses

Traditional latent variable approaches (e.g., structural equation modelling) typically focus on underlying causes, based on the idea that observable symptoms (e.g., anhedonia) can be reduced to a small set of latent causes (the disorder “itself”, i.e., depression) and they resolve once the underlying cause is treated. Based on this perspective, pairwise symptom associations are assumed to be an artefact of this shared underlying cause. In contrast, the network approach to psychopathology asserts that mental disorders intrinsically consist of the pairwise associations between symptoms (Borsboom and Cramer, 2013; Borsboom, 2017). Investigating mental disorders through the exploration of pairwise symptom associations (e.g., the pain-suicidality association) that contribute to the manifestation of broader psychopathology is consistent with statistical network models (Borsboom and Cramer, 2013).

Network models capture the statistical relationships between suicidality, pain and other known correlates of suicidality in adolescence, and their parameters can be displayed intuitively in network visualizations (Borsboom and Cramer, 2013; De Beurs, 2017). The typical visualization of such network models consists of ‘nodes’ that represent each variable entered into the network (e.g., correlates) and of ‘edges’ that represent significant pairwise relationships by connecting associated variables, with thicker edges reflecting stronger pairwise associations (Borsboom and Cramer, 2013). However, if two variables are statistically independent, then the connecting edge will be absent (Epskamp and Fried, 2018). By exploring moderation effects in moderated network models researchers can additionally learn for whom a possible pairwise association may be particularly pronounced (e.g., a pairwise association between pain and suicidality, depending on the level of depression; Haslbeck et al., 2019).

#### 2.3.3 Network estimation

Using a series of regularised network models, we scrutinised the cross-sectional pain-suicidality association, after conditioning on depression, anxiety, peer problems and inhibitory control deficits. All variables were modelled as categorical, given the skewed marginal distributions (FigS1) and to obtain categories consistent with earlier studies (Hinze et al., 2021; Kuyken et al., 2017). Given the mixture of binary and ordinal data, we estimated pairwise associations, using Mixed Graphical Models for the whole sample and both genders (see Haslbeck and Waldorp, 2020). Network models were estimated via *l_1_*-regularised [LASSO] neighbourhood regression, using the Extended Bayesian Information Criterion [EBIC] and a gamma of 0.5 (R-package *mgm*: version 1.2-10; Haslbeck et al., 2019; Haslbeck and Waldorp, 2020). Using LASSO regularisation, small and potentially spurious estimates are set to zero, leading to a sparser network graph, which is consistent with common practices (Haslbeck and Waldorp, 2018; van Borkulo et al., 2014). Network graphs were displayed using the ‘*circle*’ layout (R-package *qgraph*: version 1.6.5; Epskamp et al., 2020). Furthermore, we explored network stability across 200 bootstrap samples (R-package *mgm*, version 1.2-10; Haslbeck and Waldorp, 2020).

We computed moderated network models to investigate whether these pairwise associations depend on depression with the same nodewise-regression approach described above, including an exploration of network stability across 200 bootstrap samples (R-package *mgm*: version 1.2-10; Haslbeck and Waldorp, 2020). We conditioned on the different levels of depression and plotted the moderated network models (R-package *qgraph*: version 1.6.5; Epskamp et al., 2020). Furthermore, we explored the nodewise predictability in terms of accuracy (i.e., proportion of correct classification; Haslbeck and Waldorp, 2018). This allows us to judge how well each node can be predicted by all other nodes in the network (Haslbeck and Waldorp, 2018). We report the accuracy of the whole model, the intercept-only model and the improved predictability of a given node by all other nodes in the network (Haslbeck and Fried, 2017). Finally, we explored the expected influence of each node in the network (R-package *networktools*: version 1.2.3; Jones, 2020). *One-step* expected influence [EI1] shows how well a given node influences its direct neighbours and *two-step* expected influence [EI2] summarises a node's direct and indirect influence up to two edges away from that node (Robinaugh et al., 2016). As the current sample is representative of the general population, restricted symptom variability may bias conclusions about a node's importance, which was tested by computing Pearson correlations between the expected influence indices and the node's variance (Heeren et al., 2018; Terluin et al., 2016).

## 3 Results

### 3.1 Participant characteristics

The study sample consisted of 8072 adolescents, aged 11 to 15 years (*M*=12.6 years, *SD*=0.61), with the majority being aged 12 (45%) or 13 (48%) years. Nearly three-quarters of adolescents self-classified as white British (*n*=5967; 73.9%). More than half of participants identified as female (*n*=4380; 54.3%), mainly due to the fact that the school sample included nine female-only schools. Boys and girls differed significantly on all reported variables, including our main variables pain, suicidality and depression, except cohort and inhibitory control deficits (Table 1). We report the results of pupils with binary gender data, omitting those who selected ‘other/prefer not to say’ to the gender question (*n*=158; 2%) or had missing gender data (*n*=145; 1.8%), to avoid complexity in further gender comparisons. As the extent of missing data was low for all variables (<10% for anxiety, <4% for gender and ethnicity and <1% for all other variables; Table 1), we performed complete-case analyses, which is consistent with recommended procedures (Jakobsen et al., 2017). Table 1 provides an overview of participant characteristics.

**Table 1:** Participant characteristics (*N*=8072).

	Gender (*n*=7769; 96.3%)^a^		
	Girls (*n*=4380; 54.3%)	Boys (*n*=3389; 42.0%)	Total (*N*=8072)
***Demographics***			
Cohort			
1, *n* (%)	508 (11.6)	392 (11.6)	923 (11.4)
2, *n* (%)	3872 (88.4)	2997 (88.4)	7149 (88.6)
Age, *M* (*SD*)*	12.61 (0.61)	12.66 (0.62)	12.62 (0.61)
Ethnicity*			
White British, *n* (%)	3170 (72.4)^b^	2680 (79.1)^c^	5967 (73.9)^d^
Asian British, *n* (%)	519 (11.9)^b^	284 (8.4)^c^	819 (10.2)^d^
Black British, *n* (%)	251 (5.7)^b^	150 (4.4)^c^	407 (5.0)^d^
Arab British, *n* (%)	86 (2.0)^b^	63 (1.9)^c^	153 (1.9)^d^
Mixed Ethnic Group, *n* (%)	230 (5.3)^b^	121 (3.6)^c^	363 (4.5)^d^
Other, *n* (%)	113 (2.6)^b^	74 (2.2)^c^	191 (2.4)^d^
***Clinical Characteristics***			
Suicidality, *n* (%)*	1032 (23.6)^e^	496 (14.6)^b^	1611 (20.0)^i^
Suicidal thoughts, *n* (%)*	829 (18.9)^f^	383 (11.3)^g^	1280 (15.9)^j^
Self-harm thoughts, *n* (%)*	617 (14.1)^f^	234 (6.9)^h^	898 (11.1)^k^
Self-harm behaviours *n* (%)*	417 (9.5)^f^	153 (4.5)^h^	599 (7.4)^k^
Pain			
Pain in the past six months (SDQ; *n* (%))*	2577 (58.8)^l^	1388 (41.0)^o^	4124 (51.1)^r^
Pain on assessment day (CHU-9D; *n* (%))*	1488 (34.0)^m^	859 (25.4)^p^	2453 (30.4)^s^
Pain (both six months and todays pain combined; *n* (%))*	1196 (27.3)^n^	515 (15.2)^q^	1794 (22.2)^t^
Depression (CES-D; *M* (*SD*))*	17.62 (11.86)^u^	12.74 (9.16)^v^	15.57 (11.06)^w^
Normal, *n* (%)	2220 (50.7)^u^	2348 (69.3)^v^	4722 (58.5)^w^
At risk, *n* (%)	1244 (28.4)^u^	751 (22.2)^v^	2087 (25.9)^w^
Caseness, *n* (%)	890 (20.3)^u^	269 (7.9)^v^	1215 (15.1)^w^
Anxiety (RCADS)*			
Non-clinical, *n* (%)	3500 (79.9)^x^	2912 (85.9)^y^	6412 (79.4)^z^
Borderline, *n* (%)	231 (5.3)^x^	85 (2.5)^y^	316 (3.9)^z^
Clinical, *n* (%)	455 (10.4)^x^	144 (4.3)^y^	599 (7.4)^z^
Inhibitory Control Deficits (SDQ; *M* (*SD*))	4.28 (2.52)^l^	4.38 (2.49)^v^	4.33 (2.51)^aa^
Normal, *n* (%)	3021 (69.0)^l^	2280 (67.3)^v^	5497 (68.1)^aa^
Borderline, *n* (%)	460 (10.5)^l^	357 (10.5)^v^	855 (10.6)^aa^
High, *n* (%)	355 (8.1)^l^	292 (8.6)^v^	673 (8.3)^aa^
Very high, *n* (%)	536 (12.2)^l^	439 (13.0)^v^	1017 (12.6)^aa^
Peer Problems (SDQ; *M* (*SD*))*	2.11 (1.88)^l^	1.95 (1.84)^v^	2.06 (1.88)^aa^
Normal, *n* (%)	2904 (66.3)^l^	2321 (68.5)^v^	5401 (66.9)^aa^
Borderline, *n* (%)	587 (13.4)^l^	430 (12.7)^v^	1058 (13.1)^aa^
High, *n* (%)	367 (8.4)^l^	269 (7.9)^v^	663 (8.2)^aa^
Very high, *n* (%)	514 (11.7)^l^	348 (10.3)^v^	920 (11.4)^aa^

***Note.*** The star symbol (*) highlights significant gender differences (*p*<0.05). Proportion of missing data: anxiety (10%), gender and ethnicity (<4%) and all other variables (<1%). Participants with missing data: ^a.^*n=*303, ^b.^*n=*11, ^c.^*n=*17, ^d.^*n=*172, ^e.^*n=*7, ^f.^*n=*10, ^g.^*n=*14, ^h.^*n=*13, ^i.^*n=*18, ^j.^*n=*24, ^k.^*n=*23, ^l.^*n=*8, ^m.^*n=*18, ^n.^*n=*20, ^o.^*n=*25, ^p.^*n=*31, ^q.^*n=*37, ^r.^*n=*34, ^s.^*n=*54, ^t.^*n=*62, ^u.^*n=*26, ^v.^*n=*21, ^w.^*n=*48, ^x.^*n=*194, ^y.^*n=*248, ^z.^*n=*745, ^aa.^*n=*30.

**Fig. 2:**
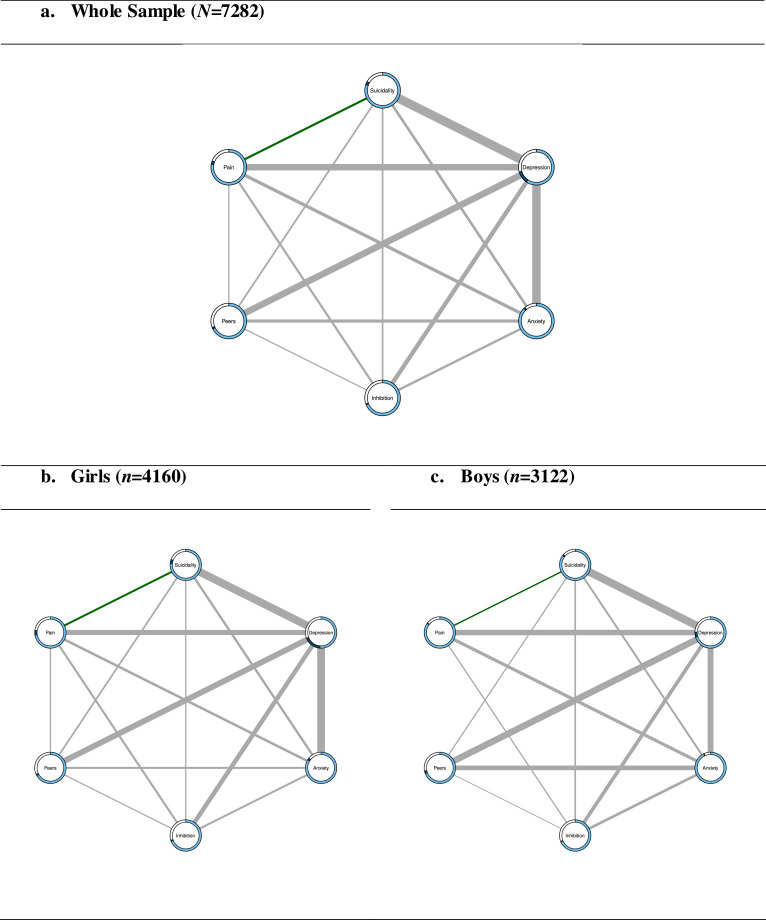
**Pairwise network models for the whole sample and both genders**. The light blue part of the rings represents the predictability by the intercept model. The dark blue part represents the additional predictability in a given node by all other nodes in the network. The sum of both blue parts reveals the predictability of the whole model. The green edge represents a positive weight between binary variables and the grey edges represent relationships between categorical variables of more than two levels, for which more than one parameter is estimated and therefore no sign can be defined. **Legend:** Inhibition=Inhibitory Control Deficits, Peers=Peer Problems, Pain=Combined pain measure.

Overall, 1794 (22.2%) adolescents reported pain (i.e., pain in the past six months *and* on the assessment day, hereafter called ‘pain’), of whom 717 (40.0%) also reported suicidality (FigS2). Of the remaining 6216 (77.0%) adolescents, who reported either only pain in the past six months *or* on the assessment day (*n*=2976; 36.9%), or no pain at all (*n*=3240; 40.1%), 889 (14.3%) adolescents reported suicidality. In total, 1611 (20.0%) adolescents reported suicidality, of whom 717 (44.5%) reported pain. Of the remaining 6443 (79.8%) adolescents without suicidality, 1075 (16.7%) reported pain. Whilst overall 717 (8.9%) adolescents reported pain and suicidality, 5326 (66.0%) adolescents reported neither pain nor suicidality.

### 3.2 The pain-suicidality association

We found a significant pain-suicidality association, showing that pain was associated with a four-fold increased risk of suicidality and vice versa (*OR*=4.00, 95%-CI=[3.54;4.51]). This cross-sectional association was significant in both girls (*OR*=3.80, 95%-CI=[3.27;4.42]) and boys (*OR*=3.45, 95%-CI=[2.75;4.31]), with no difference between the two genders. These analyses are largely consistent with the separate analyses for both pain items, showing a three-fold increased risk in the whole study sample and in both genders (Supplement 3).

We found a significant cross-sectional association between suicidality and pain for all levels of depression in the whole sample and in the subsample of girls, whilst in boys, the pain-suicidality association was only significant for those within normal depression scores (Table 2). Separate analyses for pain on the assessment day largely confirmed the findings with the exception that for boys the association was significant for all levels of depression (Sup3Table4). For pain in the past six months this association was only significant for adolescents with fewer depressive symptoms (‘normal’) in the whole sample and for girls (Sup3Table1).

**Table 2:** The pain-suicidality association by levels of depression and gender.

Moderator: Depression (*n*=8024)	Pain-Suicidality Association
Normal (*n*=4722)	OR=1.77, 95%CI=[1.23; 2.50]*
Girls (*n*=2220)	OR=1.73, 95%CI=[1.04; 2.78]*
Boys (*n*=2348)	OR=1.84, 95%CI=[1.03; 3.13]*
At Risk (*n*=2087)	OR=1.45; 95%CI=[1.18; 1.79]*
Girls (*n*=1244)	OR=1.49; 95%CI=[1.14; 1.94]*
Boys (*n*=751)	OR=1.26, 95%CI=[0.86; 1.83]
Caseness (*n*=1215)	OR=1.60; 95%CI=[1.26; 2.04]*
Girls (*n*=890)	OR=1.60; 95%CI=[1.20; 2.12]*
Boys (*n*=269)	OR=1.39, 95%CI=[0.83; 2.34]

***Note.*** The symbol ‘*’ highlights significant associations (*p*<0.05). **Legend:** Pain = Combined pain measure.

### 3.3 Network analyses for the whole study sample

**Fig. 3:**
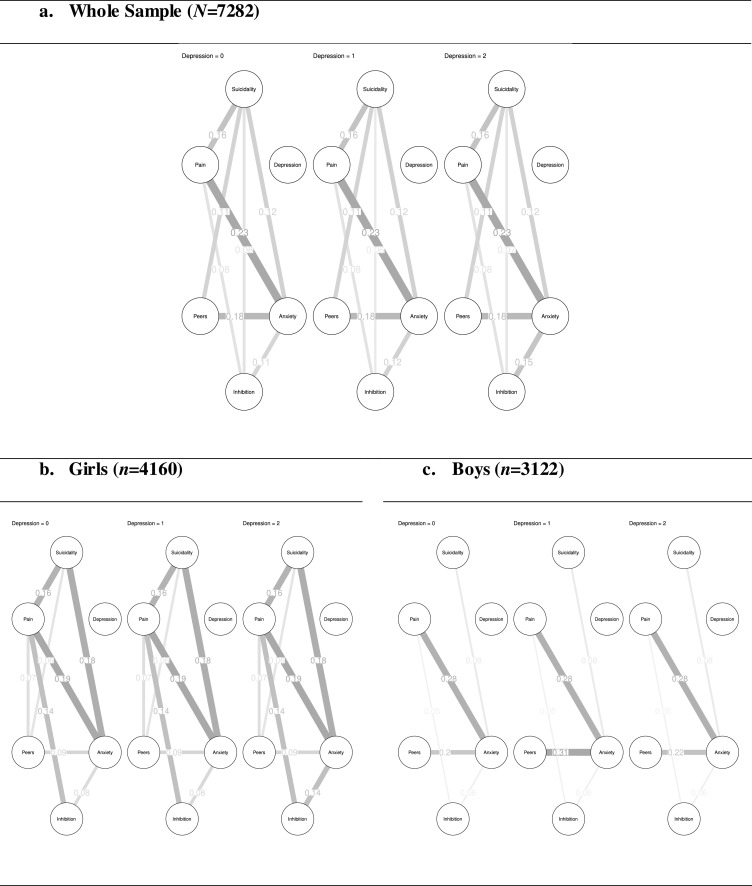
**Moderated network models conditioned on the different levels of depression**. **Legend**: 0 = ‘normal’, 1 = ‘at risk’ and 2 = ‘caseness’. Inhibition = Inhibitory control deficits, Peers = Peer problems, Pain = Combined pain measure.

The network model revealed a pairwise, cross-sectional association between pain and suicidality, after conditioning on depression, anxiety, inhibitory control deficits and peer problems (Fig. 2; weight=0.17 in Table 3; Bootstrapped 95%CI [0.10;0.22]; FigS3), showing that self-reported pain increased the probability of suicidality and vice versa (*aOR*=1.39). The whole model accuracy in predicting suicidality was 0.83 (intercept-only model=0.80), whilst for pain it was 0.81 (intercept-only model=0.78; TableS1). Suicidality and pain were associated with all nodes in the network, particularly depression (Table 3; Suicidality-Depression: Bootstrapped 95%CI [0.84;0.96]; Pain-Depression: Bootstrapped 95%CI [0.59;0.70]; FigS3). Depression was the most influential node in the network (EI1=3.45; EI2=8.42; TableS1). This finding was not influenced by restricted symptom variability (EI1: *r*=0.38, *p*=0.46). These findings are largely consistent with the separate sensitivity analyses for both pain items (Supplement 3), with the only exception that improvements in the predictability of pain by all other nodes were larger when focussing on pain in the past six months (Sup3Table2).

**Table 3:** Weights matrices for the regularised network models.

Whole Sample (*N*=7282)						
	Suicidality	Depression	Anxiety	Inhibition	Peers	Pain
Suicidality	-	**0.90**	0.22	0.11	0.12	0.17
Depression		-	0.85	0.40	0.65	0.64
Anxiety			-	0.19	0.28	0.30
Inhibition				-	0.07	0.17
Peers					-	0.07
Pain						-

Girls (*n*=4160)						
	Suicidality	Depression	Anxiety	Inhibition	Peers	Pain
Suicidality	-	**0.91**	0.23	0.09	0.15	0.18
Depression		-	0.90	0.49	0.66	0.58
Anxiety			-	0.19	0.18	0.25
Inhibition				-	0.08	0.20
Peers					-	0.10
Pain						-

Boys (*n*=3122)						
	Suicidality	Depression	Anxiety	Inhibition	Peers	Pain
Suicidality	-	**0.88**	0.1**7**	0.13	0.09	0.10
Depression		-	0.64	0.38	0.72	0.60
Anxiety			-	0.25	0.50	0.32
Inhibition				-	0.04	0.09
Peers					-	0
Pain						-

***Note****.* The strongest association with ‘Suicidality’ is highlighted in bold, and the strongest association with ‘Pain’ is underlined. **Legend**: Inhibition=Inhibitory Control Deficits, Peers=Peer Problems, Pain=Combined pain measure.

The moderated network model revealed a pairwise, cross-sectional, association between pain and suicidality (weight=0.16; Bootstrapped 95%CI [0.08;0.20]; FigS4; *aOR*=1.39) consistent with the pairwise model, but no moderation effect of depression on the pain-suicidality association (Fig. 3), which is consistent with 80% of the 200 bootstrap estimations (FigS4). These findings are consistent with the sensitivity analyses for pain in the past six months. For pain on the assessment day, depression was found to moderate the pain-suicidality association (Supplement 3).

### 3.4 Network analyses by gender

Separate network models for girls and boys showed that suicidality was associated with all nodes in the networks, with a significant pairwise, cross-sectional, association between suicidality and pain, after conditioning on depression, anxiety, inhibitory control deficits and peer problems (Fig. 2**;** Girls: weight=0.18 in Table 3, Bootstrapped 95%CI [0.09;0.26], *aOR*=1.42; Boys: weight=0.10 in Table 3, Bootstrapped 95%CI [0.00;0.23], *aOR*=1.25; FigS3). By adding the other nodes to the network, for girls the predictability of suicidality improved from 0.76 to 0.81 and of pain from 0.72 to 0.77, and for boys the predictability of suicidality improved from 0.85 to 0.87 and of pain from 0.85 to 0.86 (TableS1). These findings suggest a slightly larger contribution of the other nodes in predicting suicidality and pain in girls than in boys. For both genders, suicidality was most strongly associated with depression (Table 3; Girls: Bootstrapped 95%CI [0.81;0.97]; Boys: Bootstrapped 95%CI [0.78;0.98]; FigS3). Pain was associated with all nodes in the network for girls, and with all nodes except peer problems for boys. For both genders, pain was most strongly associated with depression (Table 3; Girls: Bootstrapped 95%CI [0.50;0.65]; Boys: Bootstrapped 95%CI [0.50;0.72]; FigS3). Depression was the most influential node in both networks (Girls: EI1=3.55, EI2=8.59; Boys: EI1=3.21, EI2=7.60; TableS1), which was not influenced by restricted symptom variability (Girls: EI1: *r*=0.35, *p*=0.49; Boys: EI1: *r*=0.15, *p*=0.77). These findings are largely consistent with the separate analyses for both pain items (Supplement 3). However, for boys, no association was revealed between suicidality and pain in the past six months (Sup3Table3).

Moderated network models revealed a pairwise, cross-sectional, association between pain and suicidality for girls (weight=0.16, Bootstrapped 95%CI [0.00;0.23]; FigS4; *aOR*=1.36), but not boys, and no moderation effect of depression on the pain-suicidality association, for both genders (Fig. 3), which is consistent with 80% of the 200 bootstrap estimations for girls and 72% of the bootstrap estimations for boys (FigS4). Whilst this finding is consistent with the sensitivity analyses for pain in the past six months, depression partially moderated the association between suicidality and pain on the assessment day in girls, but not boys (Sup3Fig4).

## 4 Discussion

This study scrutinises the cross-sectional relationship between pain and suicidality in adolescents and investigates whether this association is moderated by depression. We showed that pain was cross-sectionally associated with a four-fold increased risk of suicidality and vice versa in 11- to 15-year-olds (*OR*=4.00). Using network analyses, we demonstrated, across the whole sample and both genders, that the pain-suicidality association remained significant, after controlling for depression, anxiety, inhibitory control deficits and peer problems (*aOR*=1.39). This association was not moderated by depression. The finding that pain and suicidality were strongly associated with depression suggests that depression might mediate the pain-suicidality association in adolescence and should be explored in future longitudinal research. Moreover, pain should be considered as an independent correlate of suicidality in adolescents in future research and clinical practice.

A fifth of adolescents reported suicidality (20%) or pain (22%), which is consistent with similar studies (suicidality: 10-30%; Evans et al., 2005; pain: 11-38%; King et al., 2011). Together, these findings highlight that suicidality and pain are common experiences in a representative sample of adolescents in the UK.

As hypothesised, we found a four-fold increased risk of suicidality in adolescents who reported pain and vice versa. The pairwise, cross-sectional, association identified is larger than in a previous study, reporting a doubled risk of self-harm thoughts and behaviours in young people (aged 5-16 years) with pain and vice versa (Hinze et al., 2021). This may be explained by our focus on early adolescence, during which pain and suicidality show a marked increase in prevalence (Martin et al., 2007; Nock et al., 2013). As both conditions become more prevalent, the risk of co-occurrence becomes more likely, which underscores the importance of initiating preventative strategies in early adolescence (Gillies et al., 2018).

Consistent with our hypotheses and previous research (Hinze et al., 2021), the cross-sectional pain-suicidality association remained significant, showing an increased likelihood of suicidality in adolescents with pain and vice versa (*aOR*=1.39), after conditioning on depression, anxiety, inhibitory control deficits and peer problems, using network analysis for the whole sample, and for both genders separately. For the whole sample, the model accuracy in predicting suicidality and pain improved by only 3 points after adding all other nodes to the model, with slightly larger improvements in model accuracy for girls than for boys (suicidality: 5 vs. 2 points improvement; pain: 5 vs. 1-point improvement). Whilst the low prevalence of pain and suicidality in the study sample may partially explain these small improvements (Haslbeck and Fried, 2017), additional unmeasured variables could have improved predictability. Future research should explore a broader range of risk and resilience correlates of suicidality and pain in adolescence, whilst considering possible gender differences, given the higher prevalence rates of both conditions in girls than in boys (Hawton et al., 2012; King et al., 2011) and the gender differences in model accuracy and moderated network models, in order to enhance knowledge of correlates underpinning the pain-suicidality association in both genders. Importantly, despite the small improvements in predictability, for those adolescents who experience pain and suicidality, the pain-suicidality association may still be clinically meaningful and important.

For the whole sample and for girls, we found a significant cross-sectional pain-suicidality association for all levels of depression, whilst, for boys, this association was only significant for those within normal depression scores. This finding may reflect true gender differences in the pain-suicidality association; Adolescent boys experience depressive symptoms less often than girls (Breslau et al., 2017) and if they do, they may be less susceptible to develop concurrent feelings of pain, as they are not exposed to the same hormonal changes at the start of early adolescence that may predispose girls to develop both pain and depression (LeResche et al., 2005). In girls, these specific hormonal changes could initiate a cascade of neurobiological and cognitive changes, which may promote both pain and depression. Specifically, similar structural and functional changes have been found in brain regions of individuals with pain and depression, and likewise several neurotransmitters (e.g., serotonin, norepinephrine and glutamate) were found to play a role in both pain and depression (see Soltani et al., 2019). Hence, in girls, there may be multiple trajectories through which pain may be associated with suicidal outcomes (e.g., through the mutual maintenance relationship between pain and depression in adolescent girls (Soltani et al., 2019), as well as through the independent (potentially bidirectional) effects of pain on suicidal outcomes), whilst in boys the pain-suicidality association may be particularly driven by the more independent (potentially bidirectional) effects of pain on suicidal outcomes. Mechanisms underpinning this more independent association could involve a) the access to potentially lethal means (e.g., opioids), b) the increased capacity to enact self-harm following a habituation to pain, or c) pain- or treatment-induced feelings of hopelessness (Klonsky and May, 2015). However, we believe that the revealed gender differences may be more likely the result of the reduced power to reveal such associations. Compared to girls, boys reported lower prevalence rates of pain and suicidality, and they were less frequently classified as ‘at risk of depression’ (boys: *n*=751 vs. girls: *n*=1244) and ‘caseness’ (boys: *n*=269 vs. girls: *n*=890), leading to less power to reveal such associations for boys compared to girls. This interpretation is consistent with earlier research, suggesting that gender (and age) does not affect the pain-suicidality association in adolescents (Van Tilburg et al., 2011). To scrutinise these different interpretations, gender differences in the pain-suicidality association and potential underlying mechanisms warrant further research attention.

Suicidality and pain were most strongly associated with depression, which was the most influential node in the networks, suggesting potential mediation effects. However, depression did not moderate the pain-suicidality association in this adolescent sample, and for both genders separately, suggesting that pain may be an independent risk correlate of suicidality in adolescence.

### 4.1 Strengths

This study utilises moderated network analysis to provide novel insights into a possible moderating role of depression on the cross-sectional pain-suicidality association in adolescence. The rigorous data collection, using robust methods and validated measures, was reflected in the low proportion of missing data (<1%) for most variables, including pain and suicidality. The methodological and statistical rigor (e.g., using bootstrapping), the large sample size and the recency of the data increase our confidence in the findings and their generalisability.

### 4.2 Limitations & future research

Several limitations need to be considered. Consistent with previous population-based research (Evans et al., 2005), a minority (7%) of adolescents reported self-harm behaviours. Given these low prevalence rates, we collapsed self-harm behaviours and thoughts about suicide and self-harm into a single, binary ‘suicidality’ variable to identify adolescents, who experience suicidal distress. As emerging research suggests that suicidal ideation and behaviour may be associated with both shared and distinct risk factors (Mars et al., 2019), future research should explore at which stage of suicidal distress pain may be of particular importance.

We restricted ourselves to a composite pain measure, obtained from two questionnaires, the SDQ (Goodman, 1997) and the CHU-9D (Stevens, 2011). Whilst the SDQ inquires about frequent experiences of the most common manifestations of paediatric pain in the past six months, the item excluded other types of pain (e.g., back pain) and was not entirely pain specific as it also included ‘sickness’. Whilst the CHU-9D inquires specifically about pain on the assessment day, this question could have been interpreted as either ‘mental pain’ or ‘physical pain’. Also, the long-term functional impairment of pain remains unclear. The combination of both pain items may make the presence of recurrent or persistent pain (potentially also felt in other locations) more likely but reported pain may range in duration and severity. However, our sensitivity analyses for both separate pain items (Supplement 3), with adequate convergent validity, revealed largely similar results and increase confidence in our findings.

The use of secondary data meant that our self-report measures were well-suited to scrutinise the relationship between suicidality, pain and other correlates of suicidality at large-scale, providing the required power to detect moderation effects of moderate size in our non-clinical sample. Yet, very small moderation effects might require an even larger sample size to be recovered (Haslbeck, et al., 2019). Furthermore, the SDQ item specified headaches and abdominal pain as possible pain locations. Due to the item-based assessment these locations could not be differentiated in further analyses and the underlying causes of pain are unknown. As such it remains unclear whether it is primary pain (e.g., chronic primary headache) that may drive this association, or whether it could be any type of pain, including secondary manifestations as part of another aetiology (e.g., chronic postsurgical pain; Treede et al., 2019). Secondary analysis is often constrained by the data available. To develop a more complete understanding of which aspects of suicidality (e.g., suicidal intent, mental burden) and pain (e.g., location, causes, severity, chronicity, functional impairment) may drive this association, and which other unmeasured correlates may still account for this association (e.g., maladaptive coping in times of adversity; Cousins et al., 2015), future research should assess these aspects of pain and suicidality more thoroughly and investigate a broader range of empirically-supported risk and resilience correlates of suicidality and pain in adolescence.

We acknowledge the multi-level structure of our data, given the school-based assessment. However, as a thorough exploration of school-level predictors and heterogeneity was beyond the scope of this paper, we decided to fit network models based on regular regression analyses. Future research should explore clustering of pain and suicidality within schools.

Finally, the use of cross-sectional data, with different timescales for suicidality and pain, precludes conclusions about the direction of the effects. Longitudinal research should aim to establish clear timelines to learn more about how the relationship between pain and suicidality unfolds throughout adolescence and to investigate whether depression might mediate the pain-suicidality association.

## 5 Conclusion & clinical implications

In our representative sample of 11- to 15-year-olds, one in five adolescents reported suicidality or pain, and nine percent of adolescents reported both. Adolescents who reported pain were more likely to report suicidality and vice versa (*OR*=4.00) – a cross-sectional association which remained significant after controlling for depression, anxiety, inhibitory control deficits and peer problems (*aOR*=1.39). Depression did not moderate this association, suggesting that pain may be an independent correlate of suicidality in adolescents. Future research should use longitudinal designs to establish how this relationship develops during adolescence, with a focus on possible mechanisms (e.g., a mediation effect of depression), the direction of these effects, and aspects of suicidality (e.g., suicidal intent) and pain (e.g., causes and severity). Clinically our findings underscore the need to assess suicidality in adolescents with pain, and to ask about pain in those with suicidality, even in the absence of depressive symptoms. Necessary first steps involve increasing clinical awareness of pain as an independent correlate of suicidality in adolescence, along with training to identify and address other common precursors of suicidality (e.g., defeat & entrapment; O'Connor and Kirtley, 2018). As asking about suicidality is associated with a reduction of such thoughts and behaviours, particularly in adolescents (Blades et al., 2018), early inquiry and continuous monitoring of suicidal distress is crucial to offer timely help and support.

## Role of funding source

This research was partially supported by the Stiftung Oskar-Helene-Heim (VH); the FAZIT-Stiftung (VH); the Wellcome Trust (CC) [104908/Z/14/Z, 107496/Z/15/Z] and the Faculty of Social Sciences University of Oslo (BG). The research funders had no involvement in the study design, data collection, analysis or interpretation, as well as the writing of the report and the decision to submit for publication.

## Contributors

VH developed the study proposal under supervision of BG, TF and CC. JH and KH commented on the study plans. The MYRIAD Team has been involved in the data collection and the verification of the data. VH had full access to the dataset and performed all reported analyses. JH provided statistical advice and checked the syntax for accuracy. VH wrote the initial draft. BG, TF, CC, JH and KH contributed to the revisions, leading to the final manuscript. All authors reviewed and approved the final manuscript before submission.

We would like to acknowledge the contribution of the wider MYRIAD project team to this work. The MYRIAD Team comprises of Matthew Allwood (who has also verified the underlying data), Louise Auckland, Triona Casey, Katherine De Wilde, Eleanor-Rose Farley, Katie Fletcher, Nils Kappleman, Willem Kuyken, Suzannah Laws, Liz Lord, Emma Medlicott, Jesus Montero-Marin, Elizabeth Nuthall, Lucy Palmer, Ariane Petit, Alice Phillips, Isobel Pryor-Nitsch, Lucy Radley, Anam Raja, Jeremy Shackleford, Anna Sonley, Laura Taylor, Lucy Warriner, Mark Williams, Marc Bennett, Tim Dalgleish, Darren Dunning, Kirsty Griffiths, Rachel Knight, Maris Vainre, Saz Ahmed, Sarah-Jayne Blakemore, Blanca Piera Pi-Sunyer, Lucy Foulkes, Jovita Leung, Ashok Sakhardande, Obioha C Ukoumunne, Susan Ball, Sarah Byford, Poushali Ganguli, Mark Greenberg, Russell Viner and Brian Wainman.

These individuals have worked across the MYRIAD strategic award *‘Promoting Mental Health and Building Resilience in Adolescence: Investigating Mindfulness and Attentional Control’*, they are acknowledged as group authors in this paper for their substantial contributions to the project in accordance with the MYRIAD Dissemination Protocol. The authors would also like to thank the following for their contributions - Jennifer Harper, Daniel Brett, Jenna Parker and Cait Griffin.

## Data sharing

The syntax files will be released on the Open Science Framework upon publication. The MYRIAD pre-intervention data is available from Prof. Kuyken (willem.kuyken@psych.ox.ac.uk) upon request (release of data is subject to an approved proposal and a signed data access agreement). Data collection for later assessment waves in the MYRIAD trial is still ongoing. Hence, the presented analyses are based on a data cut taken on 15^th^ September 2020. The data may change in future publications, if participants request their data to be deleted.

## Declarations of Competing interest

None.

## Appendix Supplementary materials

Image, application 1
