# Supplementary material for: Does depression moderate the relationship between pain and suicidality in adolescence? A moderated network analysis
Source: J Affect Disord. 2021 Sep 1;292:667–77. doi: 10.1016/j.jad.2021.05.100 (PMC8323496; doi:10.1016/j.jad.2021.05.100)
Supplement: Supplementary file 1 [file mmc1.docx]

**Supplementary Material for the Manuscript:**

Does depression moderate the relationship between

pain and suicidality in adolescence?

A moderated network analysis

**Authors:**

Verena Hinze^a^, Tamsin Ford^b^, Catherine Crane^a^, Jonas M.B. Haslbeck^c^,

Keith Hawton^d,e^, The MYRIAD Team^a^ & Bergljot Gjelsvik^a,d,f^

1. Oxford Mindfulness Centre, Department of Psychiatry, University of Oxford, Oxford, UK
2. Department of Psychiatry, University of Cambridge, Cambridge, UK
3. Psychological Methods Group, University of Amsterdam, The Netherlands
4. Centre for Suicide Research, Department of Psychiatry, University of Oxford, Oxford, UK
5. Oxford Health NHS Foundation Trust, Warneford Hospital, Oxford, UK
6. Department of Psychology, University of Oslo, Oslo, Norway

**Declarations of interest:** None.

**Abbreviations:** ‘My Resilience in Adolescence’ [MYRIAD] trial

**Corresponding author:**

Ms Verena Hinze

University of Oxford, Department of Psychiatry, Warneford Lane, Oxford, OX3 7JX

Email: verena.hinze@psych.ox.ac.uk (alternatively: [verena.hinze@web.de](mailto:verena.hinze@web.de))

**Supplement 1. Recruitment strategies and additional trial information.**

Mainstream UK secondary schools (i.e., no special schools or alternative school formats) were deemed eligible for participation in the ‘My Resilience in Adolescence’ [MYRIAD] trial, if a substantive headteacher could be identified and if the latest official school inspection did not reveal an ‘inadequate’ school quality rating, in order to ensure successful trial implementation (for the pre-registered trial protocol, dated 3^rd^ June 2016, see ISRCTN ref: 86619085; Kuyken et al., 2017). Each school had to have a structure and strategy in place to be able to deliver social emotional learning or the intervention, respectively, and each school had to have an English-speaking curriculum.

The trial was promoted at educational conferences, and all eligible schools were contacted via emails and letters. To enhance generalisability of the trial’s findings, additional recruitment efforts were made to target under-represented schools, for instance, specifically targeting schools in Scotland and Wales, and independent schools. The final sample of schools was representative of UK secondary schools (for further information about the recruitment of schools see Ford et al., 2021).

Consent for study participation was obtained from headteachers, as well as at least five qualified or experienced teachers, who agreed to participate themselves and complete study measures. Study information was then provided, by the schools, to parents or caregivers of pupils in the first and second year of secondary school. Consent for the child’s completion of study measures was obtained via opt-out from the parents or caregivers. Furthermore, pupils with parental consent (i.e., not opted-out from parents), were asked for assent at the first assessment visit (T0: Baseline visit, one-year prior to the pre-intervention visit (T1)). Pupils were deemed ineligible if they could not provide informed assent and were unable to understand English. In exceptional circumstances, pupils were excluded, based on school judgement of their inability to participate (for further information about the recruitment of pupils see Ford et al., 2021).

Data for the presented analyses was collected from pupils under exam conditions, using an online portal linked to the study’s clinical trials unit. If technical difficulties occurred, pupils completed the study measures on paper, and the answers were retrospectively double entered into the same online portal. Each data collection visit was led and supervised by a member of the research team. Efforts were made to revisit schools to collect data from pupils, who were initially absent. Pupils were not reimbursed for their study participation (Ford et al., 2021). The main trial is estimated to be completed in 2021.

At baseline (T0), 26885 pupils between the ages of 11 to 14 years took part in the assessment (Ford et al., 2021). These pupils were in their first or second year of secondary school in the academic years 2016/2017 and 2017/2018 and were recruited across 85 schools in the United Kingdom (75 schools in England, 4 schools in Northern Ireland, and 3 schools in both Scotland and Wales; for a recruitment flow-chart see also Ford et al., 2021). The pre-intervention assessment (T1) was collected one year after the baseline assessment and contains data on 8072 pupils, across 84 schools, who were in the classes that were selected for continuing MYRIAD trial participation, as outlined in the pre-registered trial protocol (see ISRCTN ref: 86619085; Kuyken et al., 2017).

**Supplement 2. Additional information on study measures.**

**Anxiety**

The Revised Children’s Anxiety and Depression Scale ([RCADS]; Chorpita, Yim, Moffitt, Umemoto, & Francis, 2000) is a valid and reliable self-report questionnaire of childhood anxiety and depression (Piqueras, Martín-Vivar, Sandin, San Luis, & Pineda, 2017). We used the 38-item anxiety subscale to establish the degree of anxiety symptoms. Participants were asked to rate each item on a four-point Likert-scale, ranging from 0 (“Never”) to 3 (“Always”), with a higher score reflecting greater symptom severity (Chorpita et al., 2000). Using the official scoring syntax without mean imputation, we derived the following categories: non-clinical (T-score ≤ 64), borderline (T-score = 65-69) and clinical (T-score ≥ 70; Child First, 2016). The official scoring syntax requires the specification of gender (i.e., female and male) and grade for the computation of T-scores. Hence, we computed T-scores for all adolescents with available binary gender data, omitting those, who selected ‘other/prefer not to say’ to the gender question (*n*=158; 2%) and with missing gender data (*n*=145; 1.8%), as no scoring guidelines were available for these cases. In keeping with a recent report on the baseline school-level data (Ford et al., 2021), we coded those pupils, who were at baseline in the first year of secondary schools (i.e., Scottish year one’s and English year seven’s) consistently as ‘English year seven’s’ at baseline to use the same categorisation of schools across the UK (i.e., year seven and year eight at baseline). As the presented (pre-intervention) data was collected one year after the baseline visit, we added one year to the baseline year groups; All pupils, who were in year seven at baseline were in year eight at the pre-intervention assessment, and those who were in year eight at baseline were in year nine at the pre-intervention assessment. After obtaining consistent year groups for all schools across the UK at the pre-intervention assessment (i.e., year eight and nine), we translated the obtained year groups into US grades, on which the official scoring syntax was based. Consistent with governmental guidelines (Government UK, 2020), the UK year group eight was coded as the US grade seven and the UK year group nine was coded as the US grade eight.

**Peer Problems and Inhibitory Control Deficits**

The Strengths and Difficulties Questionnaire ([SDQ]; Goodman, 1997) is a valid and reliable screening measure of psychopathology over a period of the last six months (Goodman, 2001). Consisting of 25 questions, the SDQ covers five domains, each comprising five questions: emotional problems, conduct problems, hyperactivity and inattention, problems in peer relationships and prosocial behaviour. All participants were asked to rate these questions on a three-point scale, ranging from 0 to 2. Higher scores on the first four subscales reflect greater problems in each domain, whilst a higher score on the last subscale, reflects more prosocial behaviour (Goodman, 1997).

The peer problem subscale of the SDQ (Goodman, 1997) was used to establish the degree of peer problems. For the current analyses, we used the recommended four-band categorisation and treated ‘peer problems’ as an ordinal variable, consisting of the categories: normal (score: 0-2), borderline (score: 3), high (score: 4) and very high (score: 5-10; Youth in Mind, 2016).

The degree of inhibitory control in daily life was established using the hyperactivity-inattention subscale of the SDQ (Goodman, 1997). Here, we used the recommended four-band categorisation and treated ‘inhibitory control deficits’ as an ordinal variable, consisting of the categories: normal (score: 0-5), borderline (score: 6), high (score: 7) and very high (score: 8-10; Youth in Mind, 2016).

**FigS1. Marginal distributions of all included variables for the whole sample.**

Legend: Inhibition = Inhibitory Control Deficits, Peers = Peer Problems, Pain = Combined pain measure.

| **Suicidality in those adolescents who report pain** |  | **Pain in those adolescents who report suicidality** |
| --- | --- | --- |
|  |  |  |

**FigS2. Proportional representation of pain and suicidality, for those with suicidality and pain, respectively.**

Legend: Pain = Combined pain measure.

**Supplement 3: Sensitivity analyses, using separate measures of pain**

**PART A: Pain in the past six months**

**Prevalence rates**

Overall, 1611 (20.0%) adolescents reported suicidality, of whom 1166 (72.4%) reported pain in the past six months. In contrast, in the remaining 6443 (79.8%) adolescents without suicidality, 2956 (45.9%) reported pain in the past six months. Furthermore, of all participants, 4124 (51.1%) adolescents reported pain the past six months of whom 1166 (28.3%) reported suicidality. Of the remaining 3914 (48.5%) adolescents without pain in the past six months, 443 (11.3%) adolescents reported suicidality.

**The association between suicidality and pain in the past six months**

In whole study sample, we found a significant cross-sectional association between suicidality and pain in the past six months, showing that pain was associated with a three-fold increased risk of suicidality and vice versa (*OR*=3.09, 95%-CI=[2.74; 3.49]). This association remained significant in both girls (*OR*=3.10, 95%-CI=[2.63; 3.66]) and boys (*OR*=2.47, 95%-CI=[2.02; 3.02]), with no difference between the two genders (*p*>0.05).

When exploring the pain-suicidality association for the different levels of depression (Sup3Table1), we found a significant cross-sectional association between suicidality and pain in the past six months for those who did not report symptoms of depression.

**Sup3Table1. The pain-suicidality association by the different levels of depression.**

| **Moderator** |  | **Pain-Suicidality Association** |
| --- | --- | --- |
| **Depression (*n*=8024)** |  | **Past six months pain** |
| Normal (*n*=4722) |  | *OR*=1.55, 95%CI=[1.21; 1.99]* |
| Girls (*n*=2220) |  | *OR*=1.63, 95%CI=[1.13; 2.35]* |
| Boys (*n*=2348) |  | *OR*=1.44, 95%CI=[0.99; 2.09] |
| At Risk (*n*=2087) |  | *OR*=1.19; 95%CI=[0.96; 1.47] |
| Girls (*n*=1244) |  | *OR*=1.22; 95%CI=[0.92; 1.64] |
| Boys (*n*=751) |  | *OR*=0.96, 95%CI=[0.68; 1.36] |
| Caseness (*n*=1215) |  | *OR*=1.26; 95%CI=[0.92; 1.72] |
| Girls (*n*=890) |  | *OR*=1.26; 95%CI=[0.86; 1.85] |
| Boys (*n*=269) |  | *OR*=1.16, 95%CI=[0.63; 2.12] |

*Note.* The symbol ‘*’ highlights for which level in the moderator the pain-suicidality association was significant.

**Network analyses for the whole study sample**

The pairwise network model consisted of six nodes and 15 non-zero edges, revealing a relationship between pain in the past six months and suicidality, after conditioning on depression, anxiety, inhibitory control deficits and peer problems (weight=0.08; Sup3Fig1). Specifically, self-reported pain in the past six months increased the probability of reporting suicidality and vice versa, after conditioning on these measured correlates (*aOR*=1.18). The edge between pain and suicidality was estimated to be nonzero for 94% of the 200 bootstrap samples (Bootstrapped 95%CI [0.00; 0.15]), suggesting some instability of the results. The accuracy of the whole model in predicting suicidality was 0.83 (intercept-only model=0.80), whilst for pain it was 0.69 (intercept-only model=0.51; Sup3Table2). Furthermore, Sup3Table3 shows that suicidality and pain were associated with all other nodes in the network, most notably depression (Suicidality-Depression: Bootstrapped 95%CI=[0.85;0.97]; Pain-Depression: Bootstrapped 95%CI=[0.53; 0.64]). Depression also appeared to have the strongest influence on the network (EI1=3.41; EI2=8.30; Sup3Table3), which was not influenced by restricted symptom variability (Terluin et al., 2016), as the centrality indices did not significantly correlate with the node’s variability (EI1: *r*=0.32, *p*=0.54; Heeren et al., 2018).

The moderated network model revealed a cross-sectional pairwise association between pain and suicidality (weight=0.07, Bootstrapped 95%CI [0.00; 0.11], Sup3Fig2; *aOR*=1.15), but no moderation effect of depression on the pain-suicidality association, which was a stable finding revealed in 92% of the 200 bootstrap samples (Sup3Fig2).

**Network analyses by gender**

The separate network models for girls and boys consisted of six nodes, with 15 non-zero edges for girls and 13 non-zero edges for boys (Sup3Fig1). For girls, suicidality was associated with all nodes in the network, showing a significant pain-suicidality association, after conditioning on depression, anxiety, inhibitory control deficits and peer problems, and regularisation for weak associations (weight=0.11; *aOR*=1.25; Sup3Table3). Bootstrapping showed that the edge between pain and suicidality was estimated to be nonzero across 92% of bootstrap samples (Bootstrapped 95%CI=[0.00; 0.18]), suggesting some instability of the results. For boys, suicidality was associated with all nodes except pain. That is, the significant cross-sectional association with pain in the whole sample, reduced to non-significance in the subnetwork of boys (Sup3Table3). Specifically, for 29% of bootstrap samples, the edge between pain and suicidality was estimated to be nonzero (Bootstrapped 95%CI=[0.00; 0.12]). Whilst for girls the predictability of suicidality improved from 0.76 to 0.80 and of pain from 0.59 to 0.70, for boys, the predictability of suicidality improved from 0.85 to 0.87 and of pain from 0.59 to 0.69 by adding the other nodes to the network (Sup3Table2). The slightly larger node-related improvements in girls, suggest a larger contribution of the other nodes in predicting suicidality and pain in girls than in boys. For girls and boys, suicidality was most strongly associated with depression (Sup3Table3), which was consistently found across all bootstrap samples (Girls: Bootstrapped 95%CI=[0.81; 0.97]; Boys: Bootstrapped 95%CI=[0.78; 1.00]).

For girls, pain was associated with all nodes in the network. For boys, pain was associated with all nodes, except suicidality and peer problems (Sup3Table3). For both genders, pain was most strongly associated with depression (Sup3Table3), which was consistently found across all bootstrap samples (Girls: Bootstrapped 95%CI=[0.45; 0.60]; Boys: Bootstrapped 95%CI=[0.44; 0.63]).

An examination of the network structures showed that depression was the most influential node in both genders (Sup3Table2), which was not influenced by restricted symptom variability (Girls: EI1: *r*=0.34, *p*=0.51; Boys: EI1: *r*=0.03, *p*=0.96).

We additionally investigated whether depression moderated the cross-sectional pain-suicidality association in girls and boys, separately. For girls, the moderated network model revealed a pairwise association between pain and suicidality (weight=0.08, Bootstrapped 95%CI [0.00; 0.15], Sup3Fig2; *aOR*=1.16), but no moderation effect of depression on the pain-suicidality association, which was a stable finding revealed in 98% of the 200 bootstrap samples (Sup3Fig2). For boys, the moderated network model revealed neither a pairwise association between pain and suicidality, nor a significant moderation effect of depression on the pain-suicidality association. These findings were stable across 200 bootstrap estimations, showing the absence of a pairwise effect in 94% of bootstrap samples and the absence of a moderation effect in 86% of bootstrap samples (Sup3Fig2).

| 1. **Whole Sample (*N*=7295)** | | |  |
| --- | --- | --- | --- |
|  | | |  |
| 1. **Girls (*n*=4166)** |  | 1. **Boys (*n*=3129)** | |
|  |  |  | |
|  |  |  |  |

**Sup3Fig1. Pairwise network models for the whole sample and both genders.**

*Note.* The light blue part of the rings represents the predictability of the intercept model, whilst the dark blue part represents the additional predictability in a given node, due to all other nodes in the network. Together, the sum of both blue parts reveals the predictability of the whole model. Whilst the green edge represents a positive edge weight, the grey edges refer to relationships between categorical variables of more than two levels and are thus computed from more than one parameter. We used the *averageLayout* function to make all networks visually comparable.

Legend: Inhibition = Inhibitory Control Deficits, Peers = Peer Problems. Pain = Pain in the past six months.

**Sup3Table2. Centrality and predictability analyses.**

| **Whole Sample (*N*=7295)** | | | | | | |
| --- | --- | --- | --- | --- | --- | --- |
|  | **Suicidality** | **Depression** | **Anxiety** | **Inhibition** | **Peers** | **Pain** |
| Expected influence | | | | | | |
| One-step | 1.48 | **3.41** | 1.85 | 1.00 | 1.22 | 1.29 |
| Two-step | 5.41 | **8.30** | 6.03 | 3.22 | 4.35 | 4.30 |
| Predictability |  |  |  |  |  |  |
| Whole model | 0.83 | 0.71 | 0.89 | 0.70 | 0.69 | 0.69 |
| Intercept model | 0.80 | 0.59 | 0.88 | 0.69 | 0.68 | 0.51 |
| Improvement | 0.03 | 0.12 | 0.01 | 0.01 | 0.01 | **0.18** |
| **Girls (*n*=4166)** |  |  |  |  |  |  |
|  | **Suicidality** | **Depression** | **Anxiety** | **Inhibition** | **Peers** | **Pain** |
| Expected influence |  |  |  |  |  |  |
| One-step | 1.51 | **3.47** | 1.75 | 1.03 | 1.13 | 1.23 |
| Two-step | 5.54 | **8.33** | 5.97 | 3.38 | 4.18 | 4.04 |
| Predictability |  |  |  |  |  |  |
| Whole model | 0.80 | 0.69 | 0.85 | 0.70 | 0.68 | 0.70 |
| Intercept model | 0.76 | 0.51 | 0.84 | 0.69 | 0.67 | 0.59 |
| Improvement | 0.04 | **0.18** | 0.01 | 0.01 | 0.01 | 0.11 |
| **Boys (*n*=3129)** |  |  |  |  |  |  |
|  | **Suicidality** | **Depression** | **Anxiety** | **Inhibition** | **Peers** | **Pain** |
| Expected influence |  |  |  |  |  |  |
| One-step | 1.25 | **3.12** | 1.78 | 0.88 | 1.31 | 0.87 |
| Two-step | 4.52 | **7.08** | 5.12 | 2.73 | 4.51 | 2.98 |
| Predictability |  |  |  |  |  |  |
| Whole model | 0.87 | 0.76 | 0.93 | 0.69 | 0.72 | 0.69 |
| Intercept model | 0.85 | 0.70 | 0.93 | 0.68 | 0.69 | 0.59 |
| Improvement | 0.02 | 0.06 | 0 | 0.01 | 0.03 | **0.10** |

*Note.* Nodes with the highest centrality and improvement estimates are highlighted in bold.

Legend: Inhibition = Inhibitory control deficits, Peers = Peer problems, Pain = Pain in the past six months; Improvement = The improvement in the predictability of a given node by all other nodes in the network, beyond the ‘intercept only model’.

**Sup3Table3. Weights matrices for the regularised network models.**

| **Whole Sample (*N*=7295)** | | | | | | |
| --- | --- | --- | --- | --- | --- | --- |
|  | **Suicidality** | **Depression** | **Anxiety** | **Inhibition** | **Peers** | **Pain** |
| Suicidality | - | **0.92** | 0.23 | 0.12 | 0.13 | 0.08 |
| Depression |  | - | 0.86 | 0.38 | 0.66 | 0.59 |
| Anxiety |  |  | - | 0.19 | 0.28 | 0.29 |
| Inhibition |  |  |  | - | 0.06 | 0.24 |
| Peers |  |  |  |  | - | 0.08 |
| Pain |  |  |  |  |  | - |
| **Girls (*n*=4166)** | | | | | | |
|  | **Suicidality** | **Depression** | **Anxiety** | **Inhibition** | **Peers** | **Pain** |
| Suicidality | - | **0.92** | 0.24 | 0.09 | 0.16 | 0.11 |
| Depression |  | - | 0.91 | 0.43 | 0.68 | 0.53 |
| Anxiety |  |  | - | 0.17 | 0.19 | 0.24 |
| Inhibition |  |  |  | - | 0.05 | 0.30 |
| Peers |  |  |  |  | - | 0.06 |
| Pain |  |  |  |  |  | - |
| **Boys (*n*=3129)** | | | | | | |
|  | **Suicidality** | **Depression** | **Anxiety** | **Inhibition** | **Peers** | **Pain** |
| Suicidality | - | **0.88** | 0.18 | 0.11 | 0.08 | 0 |
| Depression |  | - | 0.66 | 0.36 | 0.71 | 0.51 |
| Anxiety |  |  | - | 0.24 | 0.49 | 0.23 |
| Inhibition |  |  |  | - | 0.04 | 0.13 |
| Peers |  |  |  |  | - | 0 |
| Pain |  |  |  |  |  | - |

*Note.* The strongest association with ‘Suicidality’ is highlighted in bold, and the strongest association with ‘Pain’ is underlined.

Legend: Inhibition = Inhibitory Control Deficits, Peers = Peer Problems, Pain = Pain in the past six months.

| 1. **Whole Sample (*N*=7295)** |  |  |
| --- | --- | --- |
| **** | | |
| 1. **Girls (*n*=4166)** |  | 1. **Boys (*n*=3129)** |
| **** |  | **** |

**Sup3Fig2. Summaries of the edge weight estimations for the moderated network models.**

*Note*. The value shows the proportion of nonzero edges across all of the 200 bootstrap estimations, whilst the black line represents the 0.05 and 0.95 quantiles of the bootstrap sampling distributions. The pairwise effects represent the stability of associations between two variables, with values near 1 representing perfect stability across all bootstrap estimations. The moderation effects represent the stability of the effect of depression on the pairwise associations, with values near 1 suggesting that across all bootstrap estimations, depression would have an effect on the pairwise association. Both effects are shown with 95% bootstrap confidence intervals. For regularised network models, the bootstrapped confidence intervals should *not* be interpreted as a hypothesis test, but only based on the width of the intervals as a measure of the stability of the results (Epskamp, Borsboom, & Fried, 2018).

Legend: 1 = Suicidality, 2 = Depression, 3 = Anxiety, 4 = Inhibitory control deficits, 5 = Peer Problems, 6 = Pain in the past six months.

**PART B: Pain on the assessment day**

**Prevalence rates**

Overall, 1611 (20.0%) adolescents reported suicidality, of whom 861 (53.5%) reported pain on the assessment day. In contrast, in the remaining 6443 (79.8%) adolescents without suicidality, 1590 (24.7%) reported pain on the assessment day. Of all participants, 2453 (30.4%) adolescents reported pain on the assessment day of whom 861 (35.1%) reported suicidality, whilst in the remaining 5565 (68.9%) adolescents without pain on the assessment day, 746 (13.4%) adolescents reported suicidality.

**The association between suicidality and pain on the assessment day**

In whole study sample, we found a significant cross-sectional association between pain on the assessment day and suicidality, showing that pain was associated with a three-fold increased risk of suicidality and vice versa (*OR*=3.50, 95%-CI=[3.12; 3.92]). This association remained significant for girls (*OR*=3.51, 95%-CI=[3.03; 4.07]) and boys (*OR*=2.98, 95%-CI=[2.43; 3.64]), with no difference between the two genders (*p*>0.05).

When exploring the cross-sectional pain-suicidality association for the different levels of depression, we found a significant association between suicidality and pain on the assessment day for all levels of depression, particularly ‘caseness’ (Sup3Table4).

**Sup3Table4. The pain-suicidality association by the different levels of depression.**

| **Moderator** |  | **Pain-Suicidality Association** |
| --- | --- | --- |
| **Depression (*n*=8024)** |  | **Pain on the assessment day** |
| Normal (*n*=4722) |  | *OR*=1.59, 95%CI=[1.19; 2.11]* |
| Girls (*n*=2220) |  | *OR*=1.43, 95%CI=[0.91; 2.19] |
| Boys (*n*=2348) |  | *OR*=1.69, 95%CI=[1.11; 2.54]* |
| At Risk (*n*=2087) |  | *OR*=1.44, 95%CI=[1.18; 1.75]* |
| Girls (*n*=1244) |  | *OR*=1.43, 95%CI=[1.10; 1.85]* |
| Boys (*n*=751) |  | *OR*=1.42, 95%CI=[1.00; 2.00]* |
| Caseness (*n*=1215) |  | *OR*=1.98, 95%CI=[1.54; 2.54]* |
| Girls (*n*=890) |  | *OR*=1.94, 95%CI=[1.44; 2.61]* |
| Boys (*n*=269) |  | *OR*=1.76, 95%CI=[1.03; 3.00]* |

*Note.* The symbol ‘*’ highlights for which level in the moderator the pain-suicidality association was significant.

**Network analyses for the whole study sample**

The pairwise network model consisted of six nodes and 15 non-zero edges, revealing a relationship between pain and suicidality, after conditioning on depression, anxiety, inhibitory control deficits and peer problems (weight=0.18; Sup3Fig3). Specifically, self-reported pain on the assessment day increased the probability of reporting suicidality and suicidality increased the probability of reporting pain on the assessment day, after conditioning on these other measured correlates (*aOR*=1.44). This cross-sectional association was stable across all bootstrap samples (Bootstrapped 95%CI=[0.12; 0.24]). The accuracy of the whole model in predicting suicidality was 0.83 (intercept-only model=0.80), whilst for pain it was 0.75 (intercept-only model=0.70; Sup3Table5). Sup3Table6 shows that suicidality and pain on the assessment day were associated with all other nodes in the network, most notably depression (Suicidality-Depression: Bootstrapped 95%CI=[0.84; 0.96]; Pain-Depression: Bootstrapped 95%CI=[0.44; 0.54]). Depression was the most influential node in the network (Sup3Table5), which was not influenced by restricted symptom variability (Terluin et al., 2016), as the centrality indices did not significantly correlate with the node’s variability (EI1: *r*=0.34, *p*=0.52; Heeren et al., 2018).

The moderated network model revealed a cross-sectional pairwise association between pain and suicidality (weight=0.14, Bootstrapped 95%CI=[0.00; 0.19]; *aOR*=1.32), as well as a moderation effect of depression on the pain-suicidality association (weight=0.08).. Sup3Fig4 shows that the association between suicidality and pain on the assessment day was stronger for those adolescents who reported the highest level of depressive symptoms (i.e., ‘caseness’), compared to those who classified as ‘normal’ or ‘at risk’ of depression. Across 200 bootstrap samples, the finding of a pairwise association between pain and suicidality was revealed in 92% of bootstrap samples, whilst the finding of a moderation effect of depression on this association was revealed in 74% of bootstrap samples, suggesting some instability of the effects (Sup3Fig5).

**Network analyses by gender**

The separate network models for girls and boys consisted of six nodes and 15 non-zero edges (Sup3Fig3). For girls and boys, suicidality was associated with all nodes in the network, showing a significant cross-sectional association between suicidality and pain on the assessment day, after conditioning on depression, anxiety, inhibitory control deficits and peer problems, and regularisation for weak associations (Girls: weight=0.18, *aOR*=1.44; Boys: weight=0.15, *aOR*=1.33; Sup3Table6). For girls, bootstrapping showed that the edge between pain on the assessment day and suicidality was estimated to be nonzero across all bootstrap samples (Bootstrapped 95%CI=[0.10; 0.26]). For boys, the edge between pain on the assessment day and suicidality was estimated to be nonzero across 92% of bootstrap samples (Bootstrapped 95%CI=[0.00; 0.24]). Whilst for girls the predictability of suicidality improved from 0.76 to 0.81 and of pain from 0.66 to 0.73, for boys, the predictability of suicidality improved from 0.85 to 0.87 and of pain from 0.75 to 0.77 by adding the other nodes to the network (Sup3Table5). These results show a slightly larger contribution of the other nodes in predicting depression, suicidality and pain in girls than in boys.

For girls and boys, suicidality was most strongly associated with depression (Sup3Table6), which was consistently found across all bootstrap samples (Girls: Bootstrapped 95%CI=[0.81; 0.97]; Boys: Bootstrapped 95%CI=[0.78; 0.97]).

For both genders, pain was associated with all nodes in the network, most notably depression (Sup3Table6), which was consistently found across all bootstrap samples (Girls: Bootstrapped 95%CI=[0.42; 0.56]; Boys: Bootstrapped 95%CI=[0.34; 0.53]).

An examination of the network structures showed that depression was the most influential node in the networks of both girls and boys (Sup3Table5), which was not influenced by restricted symptom variability (Girls: EI1: *r*=0.31, *p*=0.55; Boys: EI1: *r*=0.09, *p*=0.86).

For girls and boys, the moderated network models revealed cross-sectional pairwise associations between pain on the assessment day and suicidality (Girls: weight=0.12, Bootstrapped 95%CI=[0.00; 0.20]; *aOR*=1.23; Boys: weight=0.14, Bootstrapped 95%CI=[0.00; 0.21]; *aOR*=1.26). However, only for girls, a significant three-way interaction between pain on the assessment day, suicidality and depression was revealed (weight=0.09), with a stronger association for those adolescent girls, with higher levels of depression (‘caseness’), compared to those who classified as ‘normal’ and ‘at risk’ (Sup3Fig4). Across 200 bootstrap samples, the finding of a pairwise association between pain and suicidality was revealed in 72% of bootstrap samples for girls and 52% of bootstrap samples for boys, suggesting instability of these results in both genders (Sup3Fig5). The finding of a moderation effect of depression on the pain-suicidality association in girls was revealed in 70% of bootstrap samples, whilst the finding of no moderation effect of depression on the pain-suicidality association in boys was revealed in 62% of bootstrap samples, suggesting some instability of the results (Sup3Fig5).

| 1. **Whole Sample (*N*=7283)** | | |  |
| --- | --- | --- | --- |
| **** | | |  |
| 1. **Girls (*n*=4160)** |  | 1. **Boys (*n*=3123)** | |
| **** |  | **** | |
|  |  |  |  |

**Sup3Fig3. Pairwise network models for the whole sample and both genders.**

*Note.* The light blue part of the rings represents the predictability by the intercept model, whilst the dark blue part represents the additional predictability in a given node given all other nodes in the network. Together, the sum of both blue parts reveals the predictability of the whole model. Whilst the green edge represents a positive edge weight, the grey edges refer to relationships between categorical variables of more than two levels and are thus computed from more than one parameter. We used the *averageLayout* function to make all networks visually comparable.

Legend. Inhibition = Inhibitory Control Deficits, Peers = Peer Problems. PainToday = Pain on the assessment day.

**Sup3Table5. Centrality and predictability analyses.**

| **Whole Sample (*N*=7283)** | | | | | | |
| --- | --- | --- | --- | --- | --- | --- |
|  | **Suicidality** | **Depression** | **Anxiety** | **Inhibition** | **Peers** | **Pain** |
| Expected influence | | | | | | |
| One-step | 1.54 | **3.33** | 1.84 | 0.92 | 1.22 | 1.17 |
| Two-step | 5.41 | **8.06** | 5.90 | 3.09 | 4.25 | 3.83 |
| Predictability |  |  |  |  |  |  |
| Whole model | 0.83 | 0.71 | 0.89 | 0.70 | 0.69 | 0.75 |
| Intercept model | 0.80 | 0.59 | 0.88 | 0.69 | 0.68 | 0.70 |
| Improvement | 0.03 | **0.12** | 0.01 | 0.01 | 0.01 | 0.05 |
| **Girls (*n*=4160)** |  |  |  |  |  |  |
|  | **Suicidality** | **Depression** | **Anxiety** | **Inhibition** | **Peers** | **Pain** |
| Expected influence |  |  |  |  |  |  |
| One-step | 1.56 | **3.47** | 1.73 | 0.98 | 1.14 | 1.06 |
| Two-step | 5.56 | **8.21** | 5.85 | 3.41 | 4.11 | 3.57 |
| Predictability |  |  |  |  |  |  |
| Whole model | 0.81 | 0.69 | 0.86 | 0.70 | 0.69 | 0.73 |
| Intercept model | 0.76 | 0.51 | 0.84 | 0.69 | 0.67 | 0.66 |
| Improvement | 0.05 | **0.18** | 0.02 | 0.01 | 0.02 | 0.07 |
| **Boys (*n*=3123)** |  |  |  |  |  |  |
|  | **Suicidality** | **Depression** | **Anxiety** | **Inhibition** | **Peers** | **Pain** |
| Expected influence |  |  |  |  |  |  |
| One-step | 1.40 | **3.07** | 1.87 | 0.87 | 1.38 | 0.95 |
| Two-step | 4.74 | **7.26** | 5.32 | 2.84 | 4.69 | 3.10 |
| Predictability |  |  |  |  |  |  |
| Whole model | 0.87 | 0.76 | 0.93 | 0.69 | 0.72 | 0.77 |
| Intercept model | 0.85 | 0.70 | 0.93 | 0.68 | 0.69 | 0.75 |
| Improvement | 0.02 | **0.06** | 0 | 0.01 | 0.03 | 0.02 |

*Note.* Nodes with the highest centrality and improvement estimates are highlighted in bold.

Legend: Inhibition = Inhibitory control deficits, Peers = Peer problems, Pain = Pain on the assessment day; Improvement = The improvement in the predictability of a given node by all other nodes in the network, beyond the ‘intercept only model’.

**Sup3Table6. Weights matrices for the regularised network models.**

| **Whole Sample (*N*=7283)** | | | | | | |
| --- | --- | --- | --- | --- | --- | --- |
|  | **Suicidality** | **Depression** | **Anxiety** | **Inhibition** | **Peers** | **Pain** |
| Suicidality | - | **0.90** | 0.22 | 0.12 | 0.12 | 0.18 |
| Depression |  | - | 0.86 | 0.42 | 0.65 | 0.49 |
| Anxiety |  |  | - | 0.20 | 0.28 | 0.28 |
| Inhibition |  |  |  | - | 0.07 | 0.11 |
| Peers |  |  |  |  | - | 0.10 |
| Pain |  |  |  |  |  | - |
| **Girls (*n*=4160)** | | | | | | |
|  | **Suicidality** | **Depression** | **Anxiety** | **Inhibition** | **Peers** | **Pain** |
| Suicidality | - | **0.91** | 0.23 | 0.09 | 0.15 | 0.18 |
| Depression |  | - | 0.91 | 0.51 | 0.66 | 0.48 |
| Anxiety |  |  | - | 0.19 | 0.18 | 0.22 |
| Inhibition |  |  |  | - | 0.08 | 0.11 |
| Peers |  |  |  |  | - | 0.07 |
| Pain |  |  |  |  |  | - |
| **Boys (*n*=3123)** | | | | | | |
|  | **Suicidality** | **Depression** | **Anxiety** | **Inhibition** | **Peers** | **Pain** |
| Suicidality | - | **0.87** | 0.17 | 0.13 | 0.08 | 0.15 |
| Depression |  | - | 0.67 | 0.39 | 0.71 | 0.43 |
| Anxiety |  |  | - | 0.26 | 0.49 | 0.28 |
| Inhibition |  |  |  | - | 0.04 | 0.04 |
| Peers |  |  |  |  | - | 0.04 |
| Pain |  |  |  |  |  | - |

*Note.* The strongest association with ‘Suicidality’ is highlighted in bold, and the strongest association with ‘Pain’ is underlined.

Legend: Inhibition = Inhibitory Control Deficits, Peers = Peer Problems, Pain = Pain on the assessment day.

| 1. **Whole Sample (*N*=7283)** |  |  |
| --- | --- | --- |
| **** | | |
| 1. **Girls (*n*=4160)** |  | 1. **Boys (*n*=3123)** |
| **** |  | **** |

**Sup3Fig4. Moderated network models conditioned on the different levels of depression.**

Legend:0 = ‘normal’, 1 = ‘at risk’ and 2 = ‘caseness’; Inhibition = Inhibitory Control Deficits, Peers = Peer Problems, Pain = Pain on the assessment day.

| 1. **Whole Sample (*N*=7283)** |  |  |
| --- | --- | --- |
| **** | | |
| 1. **Girls (*n*=4160)** |  | 1. **Boys (*n*=3123)** |
| **** |  | **** |

**Sup3Fig5. Summaries of the edge weight estimations for the moderated network models.**

*Note*. The value shows the proportion of nonzero edges across all of the 200 bootstrap estimations, whilst the black line represents the 0.05 and 0.95 quantiles of the bootstrap sampling distributions. The pairwise effects represent the stability of associations between two variables, with values near 1 representing perfect stability across all bootstrap estimations. The moderation effects represent the stability of the effect of depression on the pairwise associations, with values near 1 suggesting that across all bootstrap estimations, depression would have an effect on the pairwise association. Both effects are shown with 95% bootstrap confidence intervals. For regularised network models, the bootstrapped confidence intervals should *not* be interpreted as a hypothesis test, but only based on the width of the intervals as a measure of the stability of the results (Epskamp, Borsboom, & Fried, 2018).

Legend: 1 = Suicidality, 2 = Depression, 3 = Anxiety, 4 = Inhibitory control deficits, 5 = Peer Problems, 6 = Pain on the assessment day.

| 1. **Whole Sample (*N*=7282)** |  |  |
| --- | --- | --- |
| **** | | |
| 1. **Girls (*n*=4160)** |  | 1. **Boys (*n*=3122)** |
| **** |  | **** |

**FigS3. Summaries of the edge weight estimations for the pairwise network models across 200 bootstrap samples.**

*Note*. The value shows the proportion of nonzero edges across all of the 200 bootstrap estimations, whilst the black line represents the 0.05 and 0.95 quantiles of the bootstrap sampling distributions. For regularised network models, the bootstrapped confidence intervals should *not* be interpreted as a hypothesis test, but only based on the width of the intervals as a measure of the stability of the results (Epskamp, Borsboom, & Fried, 2018).

Legend: 1 = Suicidality, 2 = Depression, 3 = Anxiety, 4 = Inhibitory control deficits, 5 = Peer Problems, 6 = Combined pain measure [Pain].

| 1. **Whole Sample (*N*=7282)** |  |  |
| --- | --- | --- |
| **** | | |
| 1. **Girls (*n*=4160)** |  | 1. **Boys (*n*=3122)** |
| **** |  | **** |

**FigS4. Summaries of the edge weight estimations for the moderated network models.**

*Note*. The value shows the proportion of nonzero edges across all of the 200 bootstrap estimations, whilst the black line represents the 0.05 and 0.95 quantiles of the bootstrap sampling distributions. The pairwise effects represent the stability of associations between two variables, with values near 1 representing perfect stability across all bootstrap estimations. The moderation effects represent the stability of the effect of depression on the pairwise associations, with values near 1 suggesting that across all bootstrap estimations, depression would have an effect on the pairwise association. Both effects are shown with 95% bootstrap confidence intervals. For regularised network models, the bootstrapped confidence intervals should *not* be interpreted as a hypothesis test, but only based on the width of the intervals as a measure of the stability of the results (Epskamp, Borsboom, & Fried, 2018).

Legend: 1 = Suicidality, 2 = Depression, 3 = Anxiety, 4 = Inhibitory control deficits, 5 = Peer Problems, 6 = Combined pain measure [Pain].

**TableS1. Centrality and predictability analyses.**

| **Whole Sample (*N*=7282)** | | | | | | |
| --- | --- | --- | --- | --- | --- | --- |
|  | **Suicidality** | **Depression** | **Anxiety** | **Inhibition** | **Peers** | **Pain** |
| Expected influence | | | | | | |
| One-step | 1.52 | **3.45** | 1.84 | 0.95 | 1.19 | 1.35 |
| Two-step | 5.52 | **8.42** | 6.02 | 3.19 | 4.30 | 4.62 |
| Predictability |  |  |  |  |  |  |
| Whole model | 0.83 | 0.71 | 0.89 | 0.70 | 0.69 | 0.81 |
| Intercept model | 0.80 | 0.59 | 0.88 | 0.69 | 0.68 | 0.78 |
| Improvement | 0.03 | **0.12** | 0.01 | 0.01 | 0.01 | 0.03 |
| **Girls (*n*=4160)** |  |  |  |  |  |  |
|  | **Suicidality** | **Depression** | **Anxiety** | **Inhibition** | **Peers** | **Pain** |
| Expected influence |  |  |  |  |  |  |
| One-step | 1.56 | **3.55** | 1.75 | 1.05 | 1.17 | 1.31 |
| Two-step | 5.69 | **8.59** | 6.04 | 3.62 | 4.28 | 4.40 |
| Predictability |  |  |  |  |  |  |
| Whole model | 0.81 | 0.69 | 0.86 | 0.70 | 0.68 | 0.77 |
| Intercept model | 0.76 | 0.51 | 0.84 | 0.69 | 0.67 | 0.72 |
| Improvement | 0.05 | **0.18** | 0.02 | 0.01 | 0.01 | 0.05 |
| **Boys (*n*=3122)** |  |  |  |  |  |  |
|  | **Suicidality** | **Depression** | **Anxiety** | **Inhibition** | **Peers** | **Pain** |
| Expected influence |  |  |  |  |  |  |
| One-step | 1.37 | **3.21** | 1.88 | 0.90 | 1.35 | 1.12 |
| Two-step | 4.86 | **7.60** | 5.43 | 2.93 | 4.75 | 3.88 |
| Predictability |  |  |  |  |  |  |
| Whole model | 0.87 | 0.76 | 0.93 | 0.69 | 0.72 | 0.86 |
| Intercept model | 0.85 | 0.70 | 0.93 | 0.68 | 0.69 | 0.85 |
| Improvement | 0.02 | **0.06** | 0 | 0.01 | 0.03 | 0.01 |

*Note.* Nodes with the highest centrality and improvement estimates are highlighted in bold.

Legend: Inhibition = Inhibitory control deficits, Peers = Peer problems, Pain = Combined pain measure; Improvement = The improvement in the predictability of a given node by all other nodes in the network, beyond the ‘intercept only model’.

**References**

Child First, 2016, April. Revised Children’s Anxiety and Depression Scale - RCADS SCORING

PROGRAMS. URL: <https://www.childfirst.ucla.edu/resources/> (accessed on 2020, April 6).

Chorpita, B.F., Yim, L.M., Moffitt, C.E., Umemoto, L.A., Francis, S.E., 2000. Assessment of

symptoms of DSM-IV anxiety and depression in children: A Revised Child Anxiety and

Depression Scale. Behav Res Ther. 38, 835–855. doi: 10.1016/s0005-7967(99)00130-8.

Epskamp, S., Borsboom, D., Fried, E.I., 2018. Estimating psychological networks and their

accuracy: A tutorial paper. Behav. Res. Methods 50, 195–212.

doi: https://doi.org/10.3758/s13428-017-0862-1.

Goodman, R., 1997. The strengths and difficulties questionnaire: A research note. J Child

Psychol. Psychiatry 38, 581–586. doi: 10.1111/j.1469-7610.1997.tb01545.x.

Goodman, R., 2001. Psychometric properties of the strengths and difficulties questionnaire. J. Am.

Acad. Child Adolesc. Psychiatry 40, 1337–1345. doi: 10.1097/00004583-200111000-00015.

Government UK, 2020. The national curriculum. URL: <https://www.gov.uk/national-curriculum>

(accessed on 2020, May 29^th^).

Heeren, A., Jones, P.J., McNally, R.J., 2018. Mapping network connectivity among symptoms of

social anxiety and comorbid depression in people with social anxiety disorder. J. Affect.

Disord. 228, 75–82. doi: 10.1016/j.jad.2017.12.003.

Ford, T., Degli Esposti, M., Crane, C., Taylor, L., Montero-Marin, J. … Kuyken W., 2021. The

Role of Schools in Early Adolescents’ Mental Health: Findings from the MYRIAD Study. J. Am. Acad. Child Adolesc. Psychiatry. doi: https://doi.org/10.1016/j.jaac.2021.02.016.

Kuyken, W., Nuthall, E., Byford, S., Crane, C., Dalgleish, T., Ford, T., … the MYRIAD team., 2017.

The effectiveness and cost-effectiveness of a mindfulness training programme in schools

compared with normal school provision (MYRIAD): study protocol for a randomised controlled trial. Trials 18, 194. doi: 10.1186/s13063-017-1917-4.

Piqueras, J.A., Martín-Vivar, M., Sandin, B., San Luis, C., Pineda, D., 2017. The Revised Child

Anxiety and Depression Scale: a systematic review and reliability generalization meta-analysis. J. Affect. Disord. 218, 153–169. doi: 10.1016/j.jad.2017.04.022.

Terluin, B., De Boer, M.R., De Vet, H.C., 2016. Differences in Connection Strength between Mental

Symptoms Might Be Explained by Differences in Variance: Reanalysis of Network Data Did Not Confirm Staging. PLoS One 11, e0155205. doi: 10.1371/journal.pone.0155205.

Youth in Mind, 2016, November 11th. Scoring the SDQ. URL:

<https://www.sdqinfo.org/py/sdqinfo/c0.py> (accessed on 2020, April 6^th^).
